# *N*,*N*,*N*′,*N*′-Tetra­methyl-*N*,*N*′-dipropyl­ethane-1,2-diaminium tetra­chlorido­cobaltate(II)

**DOI:** 10.1107/S1600536811050744

**Published:** 2011-11-30

**Authors:** Sari M. Närhi, Jatta Kostamo, Janne Asikkala, Raija Oilunkaniemi, Risto S. Laitinen

**Affiliations:** a Department of Chemistry, PO Box 3000, FI-90014 University of Oulu, Finland

## Abstract

The crystal structure of the title compound, (C_12_H_30_N_2_)[CoCl_4_], is composed of discrete (C_12_H_30_N_2_)^2+^ cations and [CoCl_4_]^2−^ anions. The asymmetric unit contains a half-cation and a half-anion. The atoms of the cation occupy general positions about an inversion centre, which is located at the midpoint of the central C—C bond. The Co atoms lie on a twofold rotation axis. The slightly distorted tetra­hedral coordination environment around the metal atom consists of two Cl atoms and their symmetry-related pairs.

## Related literature

For the synthesis and structural characterization of C_12_H_30_N_2_^2+^·Cl_2_^2−^, see: Närhi *et al.* (2011 ▶).
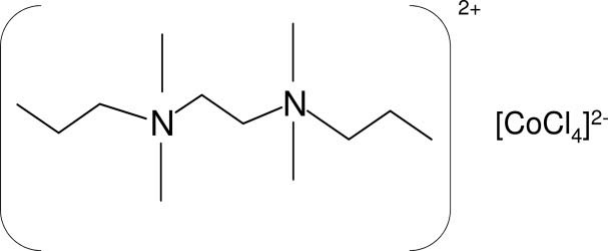


## Experimental

### 

#### Crystal data

(C_12_H_30_N_2_)[CoCl_4_]
*M* *_r_* = 403.11
Monoclinic, 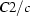

*a* = 13.583 (3) Å
*b* = 9.2334 (18) Å
*c* = 14.981 (3) Å
β = 101.83 (3)°
*V* = 1839.1 (6) Å^3^
*Z* = 4
Mo *K*α radiation
μ = 1.51 mm^−1^
*T* = 120 K
0.25 × 0.20 × 0.10 mm

#### Data collection

Bruker–Nonius KappaCCD diffractometer
Absorption correction: multi-scan (*SADABS*; Sheldrick, 2008*a* ▶) *T*_min_ = 0.705, *T*_max_ = 0.864
11798 measured reflections
1799 independent reflections
1596 reflections with *I* > 2σ(*I*)
*R*_int_ = 0.098

#### Refinement

*R*[*F* ^2^ > 2σ(*F* ^2^)] = 0.041
*wR*(*F* ^2^) = 0.107
*S* = 1.08
1799 reflections
91 parameters
H-atom parameters constrained
Δρ_max_ = 0.41 e Å^−3^
Δρ_min_ = −0.45 e Å^−3^

### 

Data collection: *COLLECT* (Bruker, 2008 ▶); cell refinement: *DENZO-SMN* (Otwinowski & Minor, 1997 ▶); data reduction: *DENZO-SMN*; program(s) used to solve structure: *SHELXS97* (Sheldrick, 2008*b* ▶); program(s) used to refine structure: *SHELXL97* (Sheldrick, 2008*b* ▶); molecular graphics: *DIAMOND* (Brandenburg, 1999) ▶; software used to prepare material for publication: *WinGX* (Farrugia, 1999 ▶).

## Supplementary Material

Crystal structure: contains datablock(s) I, global. DOI: 10.1107/S1600536811050744/hp2021sup1.cif

Structure factors: contains datablock(s) I. DOI: 10.1107/S1600536811050744/hp2021Isup2.hkl

Additional supplementary materials:  crystallographic information; 3D view; checkCIF report

## Figures and Tables

**Table 1:** Selected bond lengths (Å)

Co1—Cl1	2.2731 (9)
Co1—Cl2	2.2759 (8)

**Fig. 1.**
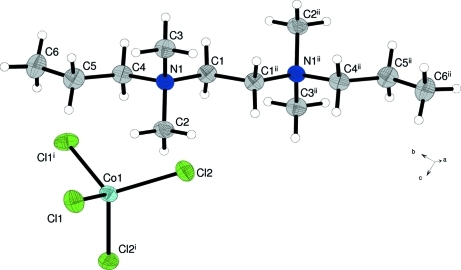
The molecular structure of the title compound indicating the numbering of the atoms. The thermal ellipsoids have been drawn at 50% probability. Symmetry code: (i) = -x, y, 0.5 - z (ii) = 0.5 - x, 0.5 - y, -z.

**Fig. 2.**
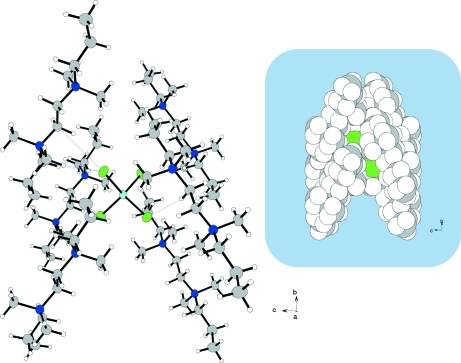
The closest contacts between an anion and the closest cations. The spacefilling presentation is shown in the insert.

**Fig. 3.**
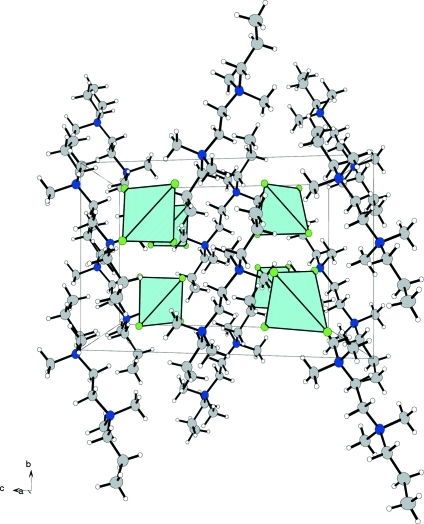
The packing of the molecules viewed along the a axis.

## supplementary crystallographic information

### Comment

The asymmetric unit of (C_12_H_30_N_2_)[CoCl_4_] consists of half of the cation and half of the anion (see Fig. 1). The N—C bond lengths in the cation range from 1.501 (3) to 1.530 (3) Å and the C—C bond lengths from 1.509 (4) to 1.525 (4) Å. These can be compared to the bond lengths in the related chloride and bromide (Närhi *et al.* 2011). In the title compound, the two *n*-propyl chains are almost coplanar with the N1—C1—C1^ii^—N1^ii^ skeleton with all torsional angles *ca* 180 °, whereas in (C_12_H_30_N_2_)Cl_2_ and (C_12_H_30_N_2_)Br_2_ the *n*-propyl chains are in the *anti*-configuration with respect to the corresponding NCCN skeleton (Närhi *et al.* 2011). The cobalt atom shows a slightly distorted tetrahedral coordination geometry and the Co—Cl bond lengths of 2.2731 (9) Å and 2.2759 (8) Å are quite normal.

The packing of the title compound consists of layers of cations. The isolated anions lay between these layers with several hydrogen bonds connecting the anions and cations, as shown in Fig. 2. The packing of the molecules is shown in Fig. 3.

### Experimental

Addition of solution of (C_12_H_30_N_2_)Cl_2_ (0.118 g, 0.432 mmol) in 5 ml MeOH to solution of CoCl_2_^.^ 6 H_2_O (0.103 g, 0.433 mmol) in 5 ml MeOH gave a purple solution from which the title compound was obtained as crystalline blue precipitate.

### Refinement

H atoms were positioned geometrically and refined using a riding model with C—H = 0.99 Å and with *U*_iso_(H) = 1.2 *U*_eq_(C) and 0.98 Å and *U*_iso_(H) = 1.2 *U*_eq_(C)for the methylene and methyl H atoms, respectively.

### Crystal data

**Table:**

(C_12_H_30_N_2_)[CoCl_4_]	*F*(000) = 844
*M**_r_* = 403.11	*D*_x_ = 1.456 Mg m^−^^3^
Monoclinic, *C*2/*c*	Mo *K*α radiation, λ = 0.71073 Å
Hall symbol: -C 2yc	Cell parameters from 1596 reflections
*a* = 13.583 (3) Å	θ = 3.1–26.0°
*b* = 9.2334 (18) Å	µ = 1.51 mm^−^^1^
*c* = 14.981 (3) Å	*T* = 120 K
β = 101.83 (3)°	Plate, blue
*V* = 1839.1 (6) Å^3^	0.25 × 0.20 × 0.10 mm
*Z* = 4	

### Data collection

**Table:**

Bruker–Nonius KappaCCD diffractometer	1799 independent reflections
Radiation source: fine-focus sealed tube	1596 reflections with *I* > 2σ(*I*)
graphite	*R*_int_ = 0.098
φ scans, and ω scans with κ offsets	θ_max_ = 26.0°, θ_min_ = 3.1°
Absorption correction: multi-scan (*SADABS*; Sheldrick, 2008*a*)	*h* = −15→16
*T*_min_ = 0.705, *T*_max_ = 0.864	*k* = −11→11
11798 measured reflections	*l* = −18→18

### Refinement

**Table:**

Refinement on *F*^2^	Secondary atom site location: difference Fourier map
Least-squares matrix: full	Hydrogen site location: inferred from neighbouring sites
*R*[*F*^2^ > 2σ(*F*^2^)] = 0.041	H-atom parameters constrained
*wR*(*F*^2^) = 0.107	*w* = 1/[σ^2^(*F*_o_^2^) + (0.0551*P*)^2^ + 2.1498*P*] where *P* = (*F*_o_^2^ + 2*F*_c_^2^)/3
*S* = 1.08	(Δ/σ)_max_ < 0.001
1799 reflections	Δρ_max_ = 0.41 e Å^−^^3^
91 parameters	Δρ_min_ = −0.45 e Å^−^^3^
0 restraints	Extinction correction: *SHELXL97* (Sheldrick, 2008), Fc^*^=kFc[1+0.001xFc^2^λ^3^/sin(2θ)]^-1/4^
Primary atom site location: structure-invariant direct methods	Extinction coefficient: 0.0131 (12)

### Special details

**Table:**

Geometry. All s.u.'s (except the s.u. in the dihedral angle between two l.s. planes) are estimated using the full covariance matrix. The cell s.u.'s are taken into account individually in the estimation of s.u.'s in distances, angles and torsion angles; correlations between s.u.'s in cell parameters are only used when they are defined by crystal symmetry. An approximate (isotropic) treatment of cell s.u.'s is used for estimating s.u.'s involving l.s. planes.
Refinement. Refinement of *F*^2^ against ALL reflections. The weighted *R*-factor *wR* and goodness of fit *S* are based on *F*^2^, conventional *R*-factors *R* are based on *F*, with *F* set to zero for negative *F*^2^. The threshold expression of *F*^2^ > 2σ(*F*^2^) is used only for calculating *R*-factors(gt) *etc*. and is not relevant to the choice of reflections for refinement. *R*-factors based on *F*^2^ are statistically about twice as large as those based on *F*, and *R*- factors based on ALL data will be even larger.

### Fractional atomic coordinates and isotropic or equivalent isotropic displacement parameters (Å^2^)

**Table:**

	*x*	*y*	*z*	*U*_iso_*/*U*_eq_	
Co1	0.0000	0.27789 (5)	0.2500	0.0254 (2)	
Cl1	0.12341 (5)	0.42242 (8)	0.32854 (5)	0.0369 (2)	
Cl2	0.06661 (5)	0.13400 (8)	0.15413 (5)	0.0348 (2)	
N1	0.24693 (16)	0.4259 (2)	0.07011 (14)	0.0271 (5)	
C1	0.2084 (2)	0.3026 (3)	0.00547 (18)	0.0289 (6)	
H1A	0.1775	0.3430	−0.0550	0.035*	
H1B	0.1556	0.2494	0.0288	0.035*	
C2	0.2940 (2)	0.3728 (3)	0.16405 (18)	0.0346 (7)	
H2A	0.3542	0.3158	0.1612	0.052*	
H2B	0.2457	0.3122	0.1874	0.052*	
H2C	0.3129	0.4559	0.2047	0.052*	
C3	0.3208 (2)	0.5173 (3)	0.03321 (19)	0.0322 (6)	
H3A	0.3404	0.6007	0.0734	0.048*	
H3B	0.2897	0.5514	−0.0280	0.048*	
H3C	0.3805	0.4595	0.0302	0.048*	
C4	0.15188 (19)	0.5122 (3)	0.07434 (18)	0.0309 (6)	
H4A	0.1194	0.5404	0.0114	0.037*	
H4B	0.1047	0.4474	0.0975	0.037*	
C5	0.1662 (2)	0.6471 (3)	0.1325 (2)	0.0379 (7)	
H5A	0.2160	0.7117	0.1130	0.046*	
H5B	0.1915	0.6209	0.1972	0.046*	
C6	0.0652 (2)	0.7244 (3)	0.1219 (2)	0.0420 (7)	
H6A	0.0388	0.7451	0.0573	0.063*	
H6B	0.0743	0.8154	0.1563	0.063*	
H6C	0.0177	0.6625	0.1454	0.063*	

### Atomic displacement parameters (Å^2^)

**Table:**

	*U*^11^	*U*^22^	*U*^33^	*U*^12^	*U*^13^	*U*^23^
Co1	0.0235 (3)	0.0307 (3)	0.0215 (3)	0.000	0.00327 (19)	0.000
Cl1	0.0295 (4)	0.0445 (5)	0.0335 (4)	−0.0022 (3)	−0.0012 (3)	−0.0116 (3)
Cl2	0.0375 (4)	0.0359 (4)	0.0341 (4)	−0.0039 (3)	0.0148 (3)	−0.0067 (3)
N1	0.0294 (11)	0.0279 (12)	0.0234 (11)	−0.0014 (9)	0.0042 (8)	−0.0016 (9)
C1	0.0251 (13)	0.0304 (14)	0.0292 (14)	−0.0022 (11)	0.0009 (10)	−0.0011 (11)
C2	0.0360 (15)	0.0416 (16)	0.0249 (13)	0.0076 (12)	0.0027 (11)	0.0036 (12)
C3	0.0308 (14)	0.0323 (15)	0.0330 (14)	−0.0063 (11)	0.0056 (11)	−0.0002 (11)
C4	0.0276 (13)	0.0348 (15)	0.0293 (14)	−0.0027 (11)	0.0037 (10)	0.0000 (11)
C5	0.0345 (15)	0.0369 (17)	0.0397 (16)	−0.0003 (12)	0.0014 (12)	−0.0016 (13)
C6	0.0363 (17)	0.0354 (17)	0.0523 (19)	0.0036 (12)	0.0044 (14)	−0.0043 (14)

### Geometric parameters (Å, °)

**Table:**

Co1—Cl1	2.2731 (9)	C2—H2C	0.9800
Co1—Cl1^i^	2.2731 (9)	C3—H3A	0.9800
Co1—Cl2	2.2759 (8)	C3—H3B	0.9800
Co1—Cl2^i^	2.2759 (8)	C3—H3C	0.9800
N1—C3	1.501 (3)	C4—C5	1.509 (4)
N1—C2	1.504 (3)	C4—H4A	0.9900
N1—C1	1.516 (3)	C4—H4B	0.9900
N1—C4	1.530 (3)	C5—C6	1.525 (4)
C1—C1^ii^	1.524 (5)	C5—H5A	0.9900
C1—H1A	0.9900	C5—H5B	0.9900
C1—H1B	0.9900	C6—H6A	0.9800
C2—H2A	0.9800	C6—H6B	0.9800
C2—H2B	0.9800	C6—H6C	0.9800
			
Cl1—Co1—Cl1^i^	108.10 (5)	N1—C3—H3A	109.5
Cl1—Co1—Cl2	108.84 (3)	N1—C3—H3B	109.5
Cl1^i^—Co1—Cl2	111.25 (3)	H3A—C3—H3B	109.5
Cl1—Co1—Cl2^i^	111.25 (3)	N1—C3—H3C	109.5
Cl1^i^—Co1—Cl2^i^	108.84 (3)	H3A—C3—H3C	109.5
Cl2—Co1—Cl2^i^	108.57 (4)	H3B—C3—H3C	109.5
C3—N1—C2	109.9 (2)	C5—C4—N1	116.4 (2)
C3—N1—C1	110.8 (2)	C5—C4—H4A	108.2
C2—N1—C1	112.2 (2)	N1—C4—H4A	108.2
C3—N1—C4	110.9 (2)	C5—C4—H4B	108.2
C2—N1—C4	109.4 (2)	N1—C4—H4B	108.2
C1—N1—C4	103.54 (19)	H4A—C4—H4B	107.3
N1—C1—C1^ii^	112.4 (3)	C4—C5—C6	108.7 (2)
N1—C1—H1A	109.1	C4—C5—H5A	110.0
C1^ii^—C1—H1A	109.1	C6—C5—H5A	110.0
N1—C1—H1B	109.1	C4—C5—H5B	110.0
C1^ii^—C1—H1B	109.1	C6—C5—H5B	110.0
H1A—C1—H1B	107.9	H5A—C5—H5B	108.3
N1—C2—H2A	109.5	C5—C6—H6A	109.5
N1—C2—H2B	109.5	C5—C6—H6B	109.5
H2A—C2—H2B	109.5	H6A—C6—H6B	109.5
N1—C2—H2C	109.5	C5—C6—H6C	109.5
H2A—C2—H2C	109.5	H6A—C6—H6C	109.5
H2B—C2—H2C	109.5	H6B—C6—H6C	109.5
			
C3—N1—C1—C1^ii^	63.7 (3)	C2—N1—C4—C5	61.9 (3)
C2—N1—C1—C1^ii^	−59.5 (3)	C1—N1—C4—C5	−178.3 (2)
C4—N1—C1—C1^ii^	−177.4 (3)	N1—C4—C5—C6	175.3 (2)
C3—N1—C4—C5	−59.4 (3)		

Symmetry codes: (i) −*x*, *y*, −*z*+1/2; (ii) −*x*+1/2, −*y*+1/2, −*z*.
